# Flexible Substrate of Cellulose Fiber/Structured Plasmonic Silver Nanoparticles Applied for Label-Free SERS Detection of Malathion

**DOI:** 10.3390/ma16041475

**Published:** 2023-02-09

**Authors:** Kseniya V. Serebrennikova, Nadezhda S. Komova, Arseniy V. Aybush, Anatoly V. Zherdev, Boris B. Dzantiev

**Affiliations:** 1 A.N. Bach Institute of Biochemistry, Research Center of Biotechnology, Russian Academy of Sciences, Leninsky prospect 33, 119071 Moscow, Russia; 2 N.N. Semenov Federal Research Center for Chemical Physics, Russian Academy of Sciences, Kosygin Street 4, 119991 Moscow, Russia

**Keywords:** Surface-enhanced Raman scattering (SERS), cellulose fiber, silver nanoparticles, silver nanostructures, silver mirror reaction, raspberry-like silver nanostructures, flexible SERS substrate, organophosphorus pesticide, malathion

## Abstract

Surface-enhanced Raman scattering (SERS) is considered an efficient technique providing high sensitivity and fingerprint specificity for the detection of pesticide residues. Recent developments in SERS-based detection aim to create flexible plasmonic substrates that meet the requirements for non-destructive analysis of contaminants on curved surfaces by simply wrapping or wiping. Herein, we reported a flexible SERS substrate based on cellulose fiber (CF) modified with silver nanostructures (AgNS). A silver film was fabricated on the membrane surface with an in situ silver mirror reaction leading to the formation of a AgNS–CF substrate. Then, the substrate was decorated through in situ synthesis of raspberry-like silver nanostructures (rAgNS). The SERS performance of the prepared substrate was tested using 4-mercaptobenzoic acid (4-MBA) as a Raman probe and compared with that of the CF-based plasmonic substrates. The sensitivity of the rAgNS/AgNS–CF substrate was evaluated by determining the detection limit of 4-MBA and an analytical enhancement factor, which were 10 nM and ~10^7^, respectively. Further, the proposed flexible rAgNS/AgNS–CF substrate was applied for SERS detection of malathion. The detection limit for malathion reached 0.15 mg/L, which meets the requirements about its maximum residue level in food. Thus, the characteristics of the rAgNS/AgNS–CF substrate demonstrate the potential of its application as a label-free and ready-to-use sensing platform for the SERS detection of trace hazardous substances.

## 1. Introduction

Surface-enhanced Raman scattering (SERS) is currently receiving much attention as a promising sensing platform for the non-destructive detection of food contaminants because of its high specificity and sensitivity [1,2]. Along with current testing techniques, including high-performance liquid chromatography (HPLC) and gas chromatography–mass spectrometry (GC-MS), SERS detection, in which a long and complex sample pretreatment is offset by extremely low detection limits, is a promising approach for detecting trace hazardous substances [3]. The enhancement of Raman scattering is due to the contribution of both electromagnetic and chemical enhancement effects. The electromagnetic mechanism is based on the local field enhancement of plasmonic nanostructures, which usually have a broad spectral band resulting in nonlinear scaling of Raman intensity [4]. It is worth noting that, in the case of the coincidence of optical excitation with a localized surface plasmon resonance for special nanostructures and configurations, so-called “hot spots” are formed, which can lead to SERS enhancement up to 10^8^ or more [5,6,7]. In contrast, the chemical-enhancement effect provided by the charge transfer mechanism between the substrate and the analyte is generally much smaller and estimated to be in the range of 10–10^3^ [8]. Much effort has been made to fabricate plasmonic substrates with a pronounced SERS effect [9]. Common SERS substrates are colloidal noble metal solutions (typically Ag, Au, and Cu) prepared with bottom-up techniques or rigid substrates fabricated using self-assembly, e-beam lithography, nanolithography, as well as the templating method [10,11]. Rigid substrates benefit over suspension systems due to prolonged storage stability and uniformity, and facilitate to a high concentration of coupled nanoparticles with a dramatically increased SERS effect [9,12]. However, the process of fabricating rigid substrates is most often complex and time-consuming [13]. In addition, the restricted compatibility of rigid substrates with sampling and the need for additional preparation procedures before analysis limits their practical application. Therefore, the fabrication of ready-to-use, SERS-active substrates in combination with portable models of Raman spectrometers [14,15] has great potential for the realization of routine analysis.

In the past few years, a new trend has appeared in the development of flexible SERS-active substrates that combine the ease and cost-effectiveness of the fabrication process with the adaptability to non-planar sample surfaces, which simplifies the sampling procedures [16,17,18,19]. To date, many flexible materials have been proposed as supports for SERS-active substrates, including cellulose preparations [20,21], adhesive tapes [22,23], flexible polymers [24,25,26], and others [27,28]. To decorate flexible materials with noble metal nanostructures, printing technology, a self-assembling technique, and lithography were successfully applied [29,30,31,32,33]. The advantages of such flexible substrates over rigid SERS substrates include simply an adjustable size and shape of the substrate, swab or wrap sampling, and tunable SERS activity that, when combined with portable Raman devices, opens up new possibilities for non-destructive testing. Currently, the pros and cons of various types of flexible SERS substrates for the detection of pesticides in fruits and vegetables [34,35,36], antibiotics in food [37,38], as well as biomarkers in human serum [39,40] were demonstrated. Despite the advances in SERS detection, the development of new reproducible SERS-active substrates with a simple and cost-effective fabrication process remains relevant for the sensitive sensing of analytes.

**Scheme 1:**
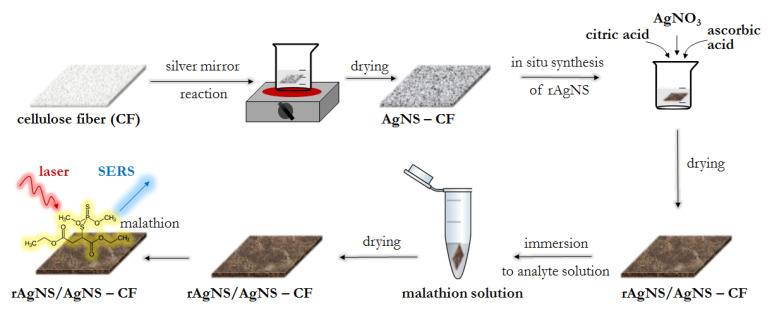
Schematic illustration of the steps for fabricating the rAgNS/AgNS–CF substrate and SERS measurement.

Since the SERS efficiency of the substrate strongly depends on the morphology of the plasmonic layer, we propose an approach for the fabrication of a flexible SERS-active substrate formed by various plasmonic silver structures on a cellulose fiber (CF) support (Scheme 1). The selection of this flexible support was based on the advantages of cellulose materials, such as wide availability, excellent flexibility, and high surface area, which promotes the high density of “hot spots” [21]. In addition, the very weak SERS response of cellulose has a low contribution to the background signal of the substrate [20]. In the first step, the CF was embedded with silver nanostructures (AgNS) as a result of an in situ silver mirror reaction to obtain an AgNS–CF substrate. In order to improve the performance of the AgNS–CF substrate, hierarchical raspberry-like silver nanostructures (rAgNS) were assembled at the second stage to obtain a rAgNS/AgNS–CF substrate. The growth of rAgNS was carried out in the presence of the AgNS–CF substrate by reducing silver nitrate with ascorbic acid without any surfactants. Hierarchical silver structures with sizes from 500 nm to 2 µm have a rough surface with a high surface density of “hot spots”, in which the SERS effect exceeds the analogous effect formed by the “hot spots” between two spherical nanoparticles [41]. Thus, the efficiency of structures with raspberry-like morphology was shown for the SERS detection of melamine adsorbed on the surface of particles [42]. In this study, the dimensional characteristics of the raspberry-like nanostructures assembled on the CF support were comparable to those in the referred study. The efficacy of the rAgNS/AgNS–CF substrate was demonstrated on the basis of a multifaceted characterization including the SERS performance for the common Raman probe 4-mercaptobenzoic acid (4-MBA). The feasibility of using the rAgNS/AgNS-CF substrate as a sensing platform for sensitive detection of malathion, an insecticide that is still used worldwide, was evaluated. This study is the first to fabricate and characterize a flexible CF-based plasmonic substrate combining the ease of fabrication and superior SERS performance afforded by silver nanostructures of various morphologies that, together with adaptability to non-planar sample surfaces and a shortened pretreatment procedure, offer great potential for pesticide detection.

## 2. Materials and Methods

### 2.1. Materials

Silver nitrate (AgNO_3_), 4-mercaptobenzoic acid (4-MBA), trisodium citrate (TSC), ascorbic acid, sodium borohydride (NaBH_4_), glucose, and citric acid were obtained from Sigma Aldrich (St. Louis, MO, USA). Ammonia solution (NH_3_) 28% and ethanol were provided by Chimmed (Moscow, Russia). Malathion was purchased from LLC ChromLab (Moscow, Russia). Milli-Q water (17.8 MΩ.cm) collected from a Milli-Q ultrapure water system (Millipore, Billerica, MA, USA) was used for the syntheses of the plasmonic nanostructures. All chemicals were of analytical grade and applied in the experiments without further purification. Cellulose fiber CFSP223000 was purchased from Millipore (Merck, Buchs, Switzerland). Before synthesis, all glassware was washed with aqua regia (HCl:HNO_3_ = 3:1) and rinsed with Milli-Q water.

### 2.2. Preparation of Plasmonic Nanostructures

#### 2.2.1. Synthesis of Ag Nanoparticles (AgNP)

Citrate-stabilized silver nanoparticles were prepared using the seed-mediated method. At the first stage, a seed solution of AgNP was synthesized as follows [43]. A total of 600 μL of sodium borohydride (6 µmol) was added to 20 mL of an aqueous solution containing both silver nitrate (5 µmol) and sodium citrate (5 µmol) with vigorous magnetic stirring. After the mixture turned bright yellow, stirring was stopped. The seed solution was left to age for 2 h. At the second stage of the synthesis [44], 10 mL of silver nitrate (10 µmol) was added to 90 mL of Milli-Q water and brought to a boil with vigorous stirring. Then, 2 mL of sodium citrate (0.3 mmol) and 4 mL of the prepared AgNP seed solution were added to synthesize the final AgNP preparation. The total volume of the solution was 106 mL. After boiling for 1 h, the colloidal suspension was cooled to room temperature and stored at 4 °C.

#### 2.2.2. Synthesis of Raspberry-like Ag Nanostructures (rAgNS)

Raspberry-like silver nanostructures were synthesized according to the previously described approach [42]. To prepare the rAgNS, 176 mg of solid ascorbic acid was rapidly added with vigorous stirring to 30 mL of an aqueous solution containing 57 mg silver nitrate and 640 mg citric acid. The formation of rAgNS occurred within 30 min as evidenced by the appearance of a gray color of the solution.

#### 2.2.3. Synthesis of Ag Nanostructures with Silver Mirror Reaction (AgNS)

In accordance with a typical silver mirror reaction to obtain AgNS [45], 10 mL of silver nitrate (0.7 mmol) was placed in a 20 mL glass flask. A total of 333 μL of sodium hydroxide (0.3 mmol) was added to the reaction mixture with vigorous stirring to form a dark brown precipitate. Then, 28% ammonium hydroxide was added dropwise until the precipitate was completely dissolved. After that, 10 mL of an aqueous glucose solution (5 mmol) was added followed by heating the mixture at 55 °C for 5 min with stirring until a silver film formed.

### 2.3. Fabrication of CF-Based Plasmonic Substrates

To compare the SERS effectiveness of the individual silver nanostructures as well as their combination on a cellulose fiber, the following plasmonic substrates were prepared in this study: CF, modified with silver nanoparticles (AgNP–CF) or raspberry-like nanostructures (rAgNS–CF); and CF, modified with silver nanostructures (AgNS–CF) and rAgNS/AgNS–CF. To fabricate the substrates of AgNP–CF, rAgNS–CF, and AgNS–CF, the pieces of CF (2 cm × 2 cm) were placed in a glass vial, and in situ synthesis of silver nanoparticles and raspberry-shaped nanostructures as well as a silver mirror reaction (see Section 2.2) were performed, respectively. Before further assembly of rAgNS, the AgNS–CF substrate was washed three times with Milli-Q water and dried at a temperature of 65 °C for 30 min in an oven. To fabricate the rAgNS/AgNS–CF substrate, the AgNS–CF substrate was placed in a reaction flask, and the synthesis of rAgNS was carried out in the presence of the substrate. Then, the as-prepared CFs were washed 3 times with Milli-Q water and left to dry at a temperature of 65 °C for 30 min in an oven.

### 2.4. Characterization of CF-Based Plasmonic Substrates

The morphologies of the as-prepared plasmonic substrates were analyzed using scanning electron microscopy (SEM). The SEM images were recorded using a scanning electron microscope (Prisma E, Thermo Fisher Scientific, Waltham, MA, USA) with accelerating voltages in the range of 2–20 kV. Energy-dispersive X-ray spectroscopy (EDX) detectors equipped with SEM (Prisma E, Thermo Fisher Scientific, USA) have yielded information about the type, abundance, and distribution of elements. The absorption spectra were measured using an UV–vis spectrophotometer (UV-3600, Shimadzu, Japan). UV–vis reflectance measurements were carried out using a Y-shaped optical fiber. The flange of the fiber was placed over the studied sample; one of the flanges was connected to the collimator (EKSMA Optics, Vilnius, Lithuania) in the front of the mercury–xenon lamp source (MKS Instruments, Inc. Andover, MA, USA), and the other was connected to the spectrometer SE2030-010-DUVN (OtO Photonics, Hsinchu, Taiwan). A sample of bare CF was used as the mirror reflection reference. Laser scanning microscope (LSM) studies were performed on the LSM-980 multicolor laser scanning microscope (Zeiss, Berlin, Germany). The measurements were made using a sensitive GaAsP PMT detector with laser powers of sub-milliwatt scale and multiple accumulations. The excitation wavelength was chosen to be 639 nm.

### 2.5. SERS Measurements

To estimate the SERS effectiveness of the fabricated CF-based plasmonic substrates, 4-MBA stock ethanol solution of standard concentrations was prepared. For the study, 10 µL of 4-MBA was dropped onto a substrate of the same size (5 mm × 5 mm) and dried in air before recording the Raman spectra.

To assess the applicability of the proposed substrate for the determination of pesticide residues, malathion–ethanol solutions of various concentrations were prepared. For analysis, the rAgNS/AgNS-CF substrate (5 mm × 5 mm) was dipped into 20 μL of a malathion standard solution. After 10 min, the substrate was removed from the solution and dried at room temperature.

SERS measurements for 4-MBA and the malathion standard solutions were accomplished on the basis of a Senterra Raman microspectrometer (Bruker, Germany) with 180º backscatter geometry and the following parameters: (1) an operating wavelength of 785 nm, which is generally suitable for the SERS of coupled and structured plasmonic nanoparticles; (2) a customized lens with a focal length of 20 cm for the efficient integration of the transverse and longitudinal heterogeneities of the CF-based substrates at the submillimeter level; (3) an acquisition time of 20 s; and (4) an output laser power of 10 mW to prevent bleaching effects on the timescale of data acquisition (bleaching curves for various output powers are presented in Figure S1). The Raman spectra were recorded at 10 randomly selected spots across the substrate. To quantify the results, the average intensity of the characteristic peak in the spectrum (signal intensity) was plotted against the analyte concentration. The SERS measurements of malathion excluded the conditions of analyte decomposition, in particular, alkaline hydrolysis of malathion [46].

## 3. Results and Discussion

### 3.1. Morphological Characterization of CF-Based Plasmonic Substrates

**Figure 1:**
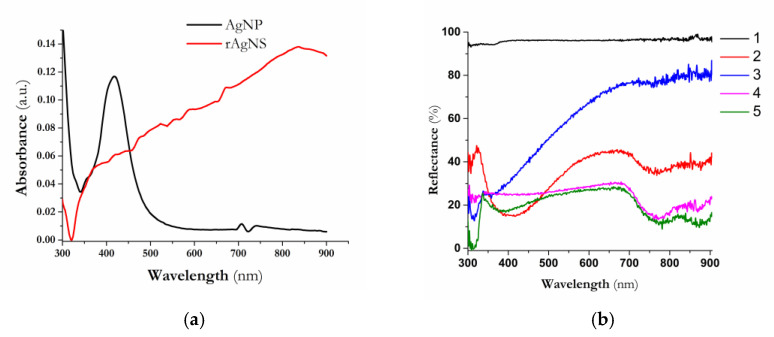
(**a**) UV–vis absorption spectra of colloidal solutions of AgNP (black curve) and rAgNS (red curve). (**b**) UV–vis reflectance spectra of bare CF (1); AgNP–CF (2); rAgNS–CF (3); AgNS–CF (4); and rAgNS/AgNS–CF (5).

The fabrication of efficient flexible SERS substrates exhibiting high electromagnetic enhancement, stability, and reproducibility is a primary task and still remains a major challenge in the development of SERS sensors. In this study, CF-based SERS substrates of different plasmonic morphologies, namely AgNP, AgNS, and rAgNS, were prepared by self-assembly via in situ synthesis and tested for SERS performance. To characterize the silver nanostructures obtained with the procedures described in the Materials and Methods section, the absorption spectra of colloidal solutions of AgNP and rAgNS in the absence of CF in the reaction medium were measured. The absorption spectra of AgNP are attributed to intraband electronic excitations of conduction electrons (plasmon oscillations) and significantly depend on the dielectric properties of the surrounding medium and the size, shape, and interaction between the nanoparticles. Smooth silver nanoparticles with a distribution ratio close to 1 have one absorption peak at approximately 400 nm and are redshifted depending on the particle size [47], which can be considered based on the Mie theory [48]; in the case of particle sizes of 40–50 nm, the peak position is usually at ~420 nm [47] for aqueous solutions (Figure 1). Additional routines are usually required to enhance the absorption efficiency in the VIS–NIR wavelength range for such AgNP. One is aimed at aggregation of nanoparticles and can consider various aspects of the classical DLVO (Deryagin–Landau–Verwey–Overbeck) theory for colloids resulting in equilibrium between van der Waals attraction and electrostatic repulsion of nanoparticles [49]. The aggregates usually contain a bunch of strongly coupled nanoparticles with the highest field enhancement factors in the gap between the nanoparticles and a fairly broad absorption shifted to the VIS–NIR range boundary. Another approach involves modifying the shape and aspect ratio of nanoparticles, which leads to the appearance of polarization-dependent plasmonic bands in different parts of the VIS range [50]. The highest field enhancement factors for this case are usually located at the edges and tips of anisotropic nanoparticles [51]. The enhancement factors provided by both edges and gaps are intrinsically combined in rAgNS, showing many “hot spots” on the surface [52]. The spectrum of rAgNS shows a very broad absorption band (Figure 1) with the contribution of multiple dipole modes of the structured surface, as well as quadrupole and higher multipole plasmon excitations due to the overall submicron size of rAgNS.

**Figure 2:**
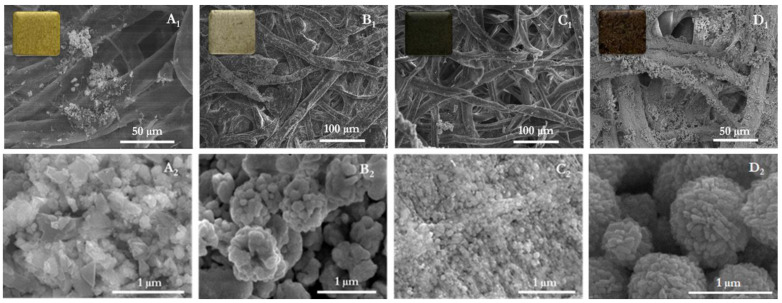
SEM images of AgNP–CF (**A_1_**,**A_2_**); rAgNS–CF (**B_1_**,**B_2_**); AgNS–CF (**C_1_**,**C_2_**); and rAgNS/AgNS–CF (**D_1_**,**D_2_**). Note: the bottom row displays magnified SEM images of the CF-based plasmonic substrates. Inserts: digital images of the as-prepared CF-based plasmonic substrates.

Figure 1b demonstrates the UV–vis reflectance spectra of the CF-based SERS substrates. The reflectance spectra of AgNP–CF, rAgNS–CF, and rAgNS/AgNS–CF showed the reflectance peaks at approximately 400 nm, which fall within the range of the characteristic surface plasmon resonance of AgNP. Modification of the CF through the silver mirror reaction forms a high-density coating that reflects on the color of the SERS substrate (insert in the Figure 2C_1_) and matches the reflection data in Figure 1b and Figure 2.

The assembly and distribution of the plasmonic nanostructures on the CF support were studied with SEM after the fabrication process of the SERS substrates, which included in situ synthesis of AgNP, AgNS, and rAgNS. The SEM images of AgNP–CF (Figure 2A_1_,A_2_) and AgNS–CF (Figure 2C_1_,C_2_) demonstrate that the surface of the CF is unevenly coated with several layers of particles. Moreover, in some areas, there is an accumulation of Ag agglomerates. The magnified image (Figure 2A_2_) shows that AgNP has an irregular and nonspherical morphology. On the contrary, a coating with densely packed AgNS is observed for AgNS–CF (Figure 2C_2_). Obviously, this fabrication technique contributes to an increase in the surface roughness of the plasmonic substrate. The SEM images of the rAgNS-decorated substrates are shown in Figure 2B_1_,B_2_,D_1_,D_2_. Comparison of these SEM images shows that, when raspberry-like nanostructures are assembled on the AgNS-–CF substrate, an essentially denser coating is formed with a highly rough surface characterized by many protrusions and naturally formed slots.

**Figure 3:**
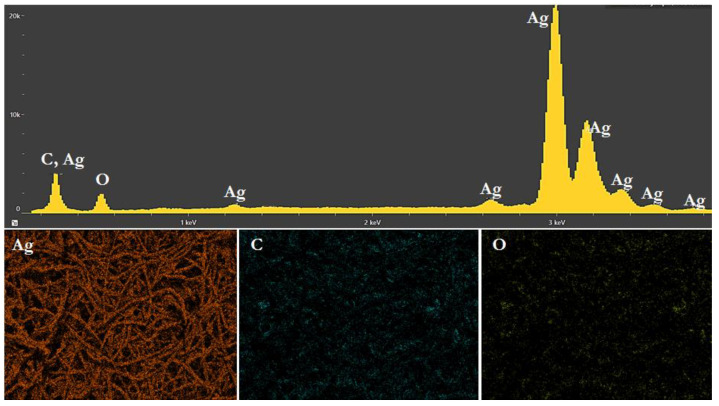
EDX spectrum and elemental mapping of Ag, C, and O acquired for rAgNS/AgNS–CF.

To characterize the composition and structure of the rAgNS/AgNS–CF substrate, elemental mapping of the substrate was implemented using EDX. Figure 3 shows the presence of elements C, O, and Ag, which correspond to the chemical composition of the substrate. Thus, it can be argued that the silver nanostructures are successfully embedded in the CF support. At the same time, the SEM image of the substrate shows that the silver nanostructures are uniformly and densely distributed over the CF, which contributes to the formation of the SERS “hot spots”.

### 3.2. SERS Performance of CF-Based Plasmonic Substrates

The SERS performance of the substrates was estimated using 4-MBA as a standard Raman probe. For the study, the Raman spectra of 4-MBA (10^–5^ M) were recorded on various CF-based plasmonic substrates under the same conditions, including laser power and acquisition time. The strong peak at 1068 cm^−1^, attributed to the aromatic ring breathing vibrations of 4-MBA, was used throughout this study. To compare the SERS effect of the as-prepared substrates, analytical enhancement factors (AEF) were calculated using the following expression:

$$AEF=\frac{I_{SERS\;}\times C_{RS}}{I_{RS}\times C_{SERS}}$$

where C_RS_ and C_SERS_ are the concentrations of 4-MBA that produce the Raman and SERS signals, respectively, while I_RS_ and I_SERS_ are the integrated intensities of the normal (spontaneous) Raman and SERS signals of 4-MBA, respectively.

**Figure 4:**
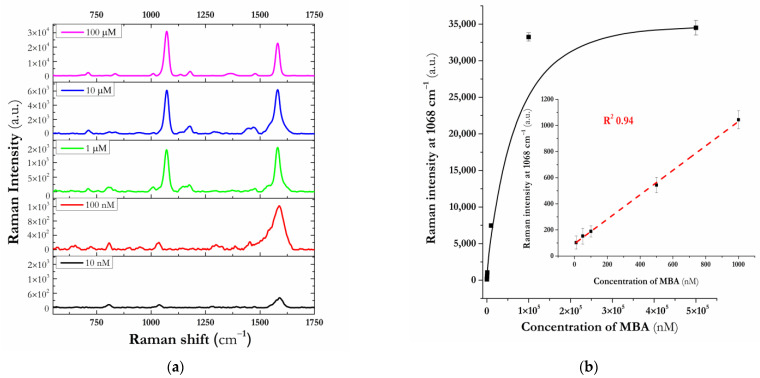
(**a**) SERS spectra of 4-MBA with different concentrations from 10^−8^ to 10^−4^M acquired on the rAgNS/AgNS–CF substrate at an excitation wavelength of 785 nm. (**b**) The dependence of the SERS signal at 1068 cm^−1^ on the 4-MBA concentration. The data are fitted using the Langmuir isotherm. The error bars represent the standard deviation of 10 measurements. Insert: linear dependence of the SERS signal in the range of 4-MBA concentrations 10^−8^–10^−6^M.

Here, C_RS_ probed in the normal Raman spectrum on the untreated CF was 10^−3^ M. Table 1 summarizes the calculated AEF for the different substrates. The uneven and sparse distribution of plasmonic AgNP and rAgNS observed in the SEM images is confirmed by the low AEFs obtained for the AgNP–CF and rAgNS–CF substrates. The AEF for rAgNS/AgNS–CF shows an order of magnitude enhancement compared to the AgNS–CF substrate (see Table 1), which can be explained by dense loading of rAgNS on the rAgNS/AgNS–CF substrates combining both enhancement factors of single and coupled hierarchical raspberry-like nanostructures. For further sensitivity evaluation of the rAgNS/AgNS–CF substrate, SERS spectra of a series of 4-MBA concentrations were obtained. Figure 4A shows the averaged (N = 10) Raman spectra of 4-MBA in a concentration range of 10^−8^–10^−4^ M. As follows from Figure 4B, the concentration dependence of the SERS characteristic peak at 1068 cm^−1^ is approximated by the fitting dependence of the Langmuir isotherm.

However, in the range of 10^−8^–10^−6^ M, the dependence between the SERS signal and the concentration of the analyte is linear and is described with the equation: $I_{SERS}=0.94+94.8\times C_{MBA}$ with R^2^ 0.94. The detection limit of 4-MBA was 10 nM.

**Figure 5:**
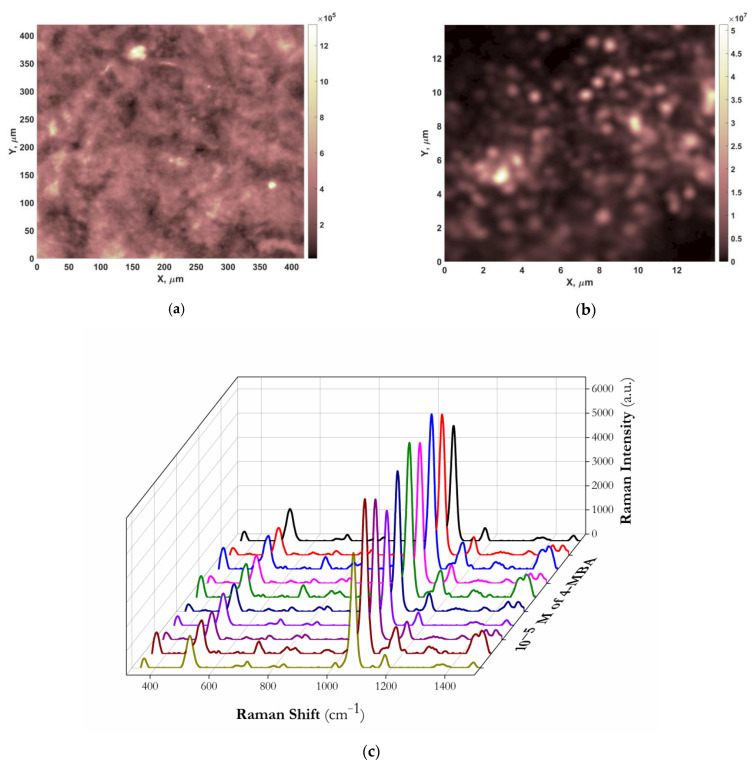
Macro (**a**) and micro (**b**) scale LSM images of 4-MBA at 1068 cm^−1^ band for rAgNS/AgNS–CF. (**c**) SERS spectra of 4-MBA measured at 10 different spots on the rAgNS/AgNS–CF substrate.

The uniformity and reproducibility of the SERS signal on the rAgNSs/AgNSs–CF substrate are important parameters for quantitative SERS detection. For CF-based substrates, which are porous multilayer structures with characteristic fiber diameters of ~20 μm, sufficient reproducibility of the Raman signal can be achieved using relatively long-focus optics and taking into account material irregularities. Figure 5a represents a macroscale LSM image of the 1068 cm^−1^ 4-MBA band for the rAgNS/AgNS–CF substrate, which combines all CF layers involved in SERS. The image shows that submillimeter scale integration is usually required. A fairly dense distribution of “hot spots” of the same 4-MBA band on the microscale is shown in Figure 5b. The characteristic dimensions of the high intensity areas, estimated within 400 nm, do not exceed the optical resolution of the image, which corresponds to the submicron size of rAgNS determined with SEM measurements (Figure 2). Thus, it can be assumed that the structured surface of rAgNS and the localization of these structures contribute to the maximum SERS enhancement factors of the substrate. The scale of the enhancement factors corresponding to the AEFs is given in Table 1. Further, the reproducibility of the Raman signal was evaluated by recording the SERS spectra at different sites of the substrate containing 4-MBA at a concentration of 10^−5^ M. As follows from Figure 5C, the intensity of the peak at 1068 cm^−1^, measured in 10 spots of the substrate, demonstrates the signal deviation within 18%. The observed variation of intensity can also be associated with a change in the orientation of the benzene ring of 4-MBA at different spots of the plasmonic substrate. Storage of the rAgNS/AgNS–CF substrate containing 10^−5^ M 4-MBA under standard laboratory conditions (T = 20 °C and humidity approximately 40%) for 2 weeks showed a decrease in the intensity of the peak at 1068 cm^−1^ by no more than 10%.

### 3.3. Application of rAgNS/AgNS–CF Substrate for SERS Detection of Malathion

To further evaluate the applicability of the proposed rAgNS/AgNS–CF substrate, malathion was tested. Malathion is one of the most common organophosphate pesticides used in pest control [53,54]. Pesticide residues on crops can cause harmful effects on the central and peripheral nervous system when ingested [55]. Therefore, the relevance of the development of reliable, selective, and sensitive sensing systems for malathion detection is obvious.

**Figure 6:**
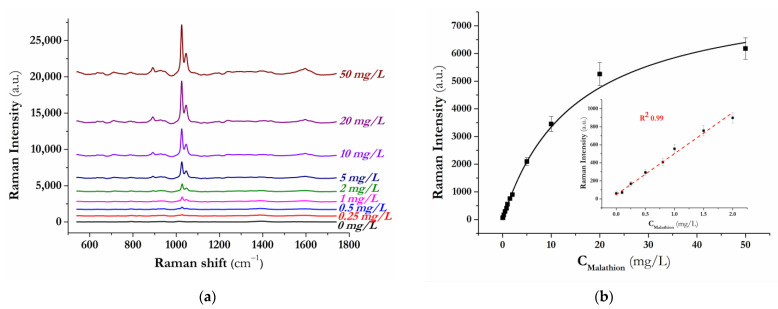
(**a**) SERS spectra of various malathion concentrations obtained on the rAgNS/AgNS–CF substrate. (**b**) The dependence of the SERS signal at 1030 cm^−1^ on the malathion concentration. The data are fitted using the Langmuir isotherm. The error bars represent the standard deviation of 10 measurements. Insert: linear dependence of the SERS signal in the range of malathion concentrations of 0.1–2 mg/L.

Prior to testing malathion, the Raman spectrum of a blank rAgNS/AgNS–CF substrate was recorded. As can be seen in Figure S2a, the blank substrate exhibits very weak scattering characteristics and no spectral interference for the SERS measurements of malathion. The immersion technique used to characterize the sensing performance of the rAgNS/AgNS–CF substrate has been described in a number of works [56,57] and simulates the analysis of real samples. The structure of malathion, consisting of fragments of O,O-dimethylthiophospho- and thiobutanedioate moieties linked by a phosphorus–sulfur bond, as well as the Raman and SERS spectra acquired on a Raman Grade CaF_2_ window and rAgNS/AgNS–CF, respectively, are shown in Figure S2b. As can be seen, the SERS spectrum undergoes significant changes compared to the Raman spectrum measured on the CaF_2_ window. The shifts of the spectral bands and the enhancement of individual peaks are explained by the proximity of the analyte to the surface of the plasmonic nanoparticles [58]. To evaluate the performance of the flexible rAgNS/AgNS–CF substrate, the SERS spectra of malathion standard solutions with concentrations from 0.25 to 50 mg/L were recorded. Figure 6a shows three strong characteristic peaks at 895 cm^−1^, 1030 cm^−1^, and 1050 cm^−1^, which can be assigned as C–C stretching, P–O–C vibration, and P=O stretching, respectively. The intensity of all characteristic peaks varied depending on the concentration of the analyte. However, the most pronounced change in intensity was observed for the dominant peak at 1030 cm^−1^, which made it possible to detect the lowest concentrations of malathion. Therefore, the characteristic peak at 1030 cm^−1^ was used for the quantitative analysis of malathion. As seen in Figure 6b, the curve of SERS intensity versus malathion concentration also follows the non-linear Langmuir isotherm fit. The non-linear equation was calculated as:

$$I_{SERS}=\frac{8295.9\times C_{malathion}\;}{C_{malathion}+14.8}$$

At low analyte levels from 0.1 to 2 mg/L, there is a good linear relationship between peak intensity and malathion concentration (insert in Figure 6b), which is described with the following equation:

$$I_{SERS}=46.9+454\times C_{malathion}$$

The detection limit was estimated to be 0.15 mg/L. Various SERS substrates applied for the detection of malathion are compared in Table 2. Here, the proposed flexible SERS substrate demonstrates the comparable performance. Moreover, it is important to note the advantages of the rAgNS/AgNS–CF substrate, such as flexibility, ease of fabrication, and material porosity, which ensure a high sorption capacity and, accordingly, a low analyte detection limit. As a whole, the mentioned benefits make the current study particularly attractive for potential practical applications in pesticide detection. According to the Food and Drug Administration (FDA) and the U.S. Environmental Protection Agency (EPA), the maximum residue limit for malathion in food is 8 mg/L [59]. Despite the high sensitivity of colorimetric [60,61,62] and electrochemical [63,64] sensors based on a specific aptamer–pesticide binding, the characteristic of the proposed label-free, flexible substrate-based SERS sensor meets the established requirements for the control of malathion in food.

**Figure 7:**
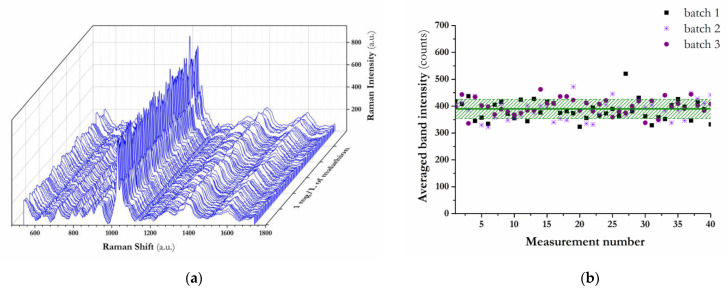
(**a**) SERS spectra of 1 mg/L malathion obtained from spot-to-spot detection. (**b**) Reproducibility of the SERS signal at 1030 cm^−1^ obtained from batch-to-batch measurements. The SERS spectra were obtained in 40 random spots of the substrate. RSD was obtained for 120 measurements of the SERS signal at 1030 cm^−1^ from 3 batches of the substrate.

To further assess the reproducibility and uniformity of the signal from the rAgNS/AgNS–CF substrate treated with the malathion at a concentration of 1 mg/L, the spectra were measured in spot-to-spot and batch-to-batch modes. First, the spectra were obtained in 40 randomly selected spots on the same substrate. Figure 7a reflects the good spot-to-spot reproducibility of the SERS response. Then, the SERS spectra acquired on different substrates made in three batches were studied. On each rAgNS/AgNS–CF substrate, 40 measurements were made in random spots. The intensity of the characteristic peak at 1030 cm^−1^ was used to calculate the relative standard deviation of 120 measurements. As follows from Figure 7b, combining silver nanostructures of different morphology on a flexible CF support provides good SERS signal homogeneity with a relative standard deviation estimated at 8.71%.

**Figure 8:**
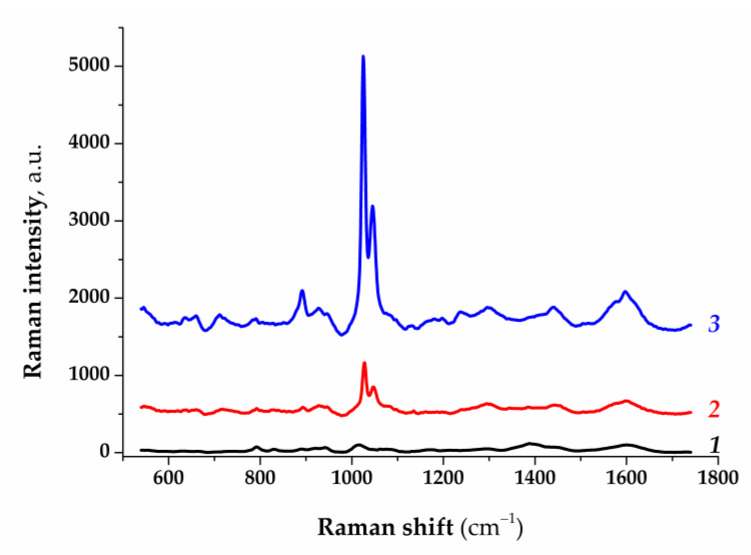
SERS spectrum of the swab collected from tomato peel (1) and SERS spectra of swabbed tomato peel spiked with 2 mg/L (2) and 20 mg/L (3) malathion standard solutions.

To further evaluate the applicability of the prepared flexible SERS substrate as a label-free sensing platform for the detection of malathion, pesticide-contaminated tomatoes were prepared according to procedure [57] with slight modifications. Ethanol solutions of malathion at concentrations of 2 and 20 mg/L were applied to the tomato peel. After complete drying at room temperature, the tomato peel was thoroughly swabbed using a 5 mm × 5 mm rAgNS/AgNS–CF substrate previously soaked in ethanol. Figure 8 shows the SERS spectra of the swabbed tomato peel without malathion, as well as in the presence of spiked malathion standard solutions. The presence of characteristic malathion peaks in the SERS spectra indicates that the proposed SERS substrate has practical potential for detecting pesticide residues.

## 4. Conclusions

In summary, a novel flexible SERS-active substrate was fabricated by successive syntheses of silver nanostructures of various morphologies. The dense coating of the CF substrate with hierarchical silver nanostructures naturally forming narrow gaps provides excellent SERS enhancement. Thus, high sensitivity with a detection limit of 10 nM was achieved for the determination of 4-MBA. The feasibility of the rAgNS/AgNS–CF substrate for practical application was demonstrated with the SERS detection of malathion with a limit of detection well below the maximum residual limits established by the protection agencies. Further research on the CF-based plasmonic substrates will be aimed at testing the versatility of the SERS sensor to various pesticide residues, as well as the possibility of multiplex detection of common organophosphorus pesticides in fruits and vegetables. Taken together, a simple and convenient fabrication process along with high sensitivity and reproducibility provide a high potential of the proposed flexible SERS substrate for practical application in label-free, non-destructive determination of pesticide residues.

## Figures and Tables

**Table 1:** AEFs calculated for different CF-based plasmonic substrates.

CF-Based Plasmonic Substrate	Analytical Enhancement Factor
AgNP–CF	1.3 × 10^3^
rAgNS–CF	4.9 × 10^3^
AgNS–CF	2.7 × 10^5^
rAgNS/AgNS–CF	8.0 × 10^6^

**Table 2:** Comparison of different plasmonic substrates for SERS detection of malathion.

SERS Substrate	Enhancement Factor	Detection Limit of Malathion, mg/L	References
Colloid of AgNP	1.98 × 10^8^	1 × 10^−4^	[65]
Colloid of AgNP modified with spermine	N/D	~0.2	[66]
Colloid of gold nanoparticles	N/D	0.123	[67]
AgNP-coated filter membrane	3.0 × 10^5^	0.062	[68]
Colloid of AgNP	N/D	0.528	[69]
Poly-(dimethylsiloxane) chip with quasi-3D gold plasmonic nanostructure arrays	1.6 × 10^6^	0.001	[70]
PDMS modified with AgNP	1.64 × 10^5^	0.041	[71]
rAgNS/AgNS-CF	8.0 × 10^6^	0.15	This study

## Data Availability

Data are presented in the article. Initial instrumental output data are available upon request from corresponding author.

## Supplementary Materials

The following supporting information can be downloaded at: https://www.mdpi.com/article/10.3390/ma16041475/s1, Figure S1: Normalized Raman Intensity at 1068 cm^−1^ versus excitation time using 10 mW (1), 25 mW (2), and 50 mW (3) laser power; Figure S2: Raman spectrum of 1g/L malathion measured on a Raman Grade CaF_2_ window (1). Raman spectrum of 1 mg/L malathion, measured on the rAgNS/AgNS–CF substrate (2). Insert: the structure of malathion.
