# Supplementary material for: Flexible Substrate of Cellulose Fiber/Structured Plasmonic Silver Nanoparticles Applied for Label-Free SERS Detection of Malathion
Source: Materials (Basel). 2023 Feb 9;16(4):1475. doi: 10.3390/ma16041475 (PMC9963878; doi:10.3390/ma16041475)
Supplement: Supplementary file 1 [file materials-16-01475-s001.zip › materials-2089012-supplementary.pdf]

Supplementary Materials

Flexible Substrate of Cellulose Fiber/Structured Plasmonic Silver Nanoparticles Applied for Label-Free SERS Detection of Malathion

Kseniya V. Serebrennikova [1], Nadezhda S. Komova [1], Arseniy V. Aybush [2], Anatoly V. Zherdev [1] and Boris B. Dzantiev [1,*]

[1] A.N. Bach Institute of Biochemistry, Research Center of Biotechnology, Russian Academy of Sciences, Leninsky prospect 33, 119071 Moscow, Russia
[2] N.N. Semenov Federal Research Center for Chemical Physics, Russian Academy of Sciences, Kosygin Street 4, 119991 Moscow, Russia
* Correspondence: dzantiev@inbi.ras.ru

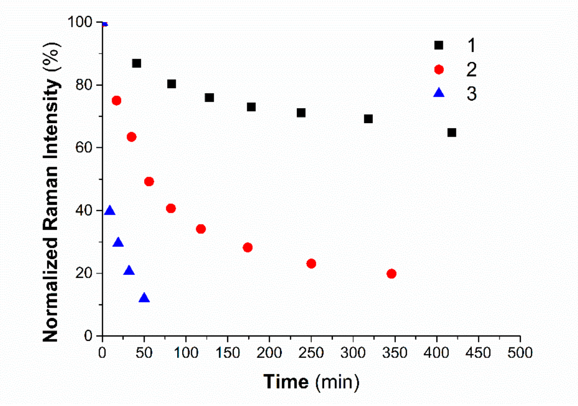

Figure S1. Normalized Raman Intensity at 1068 cm^{-1} versus excitation time using 10 mW (1), 25 mW (2) and 50 mW (3) laser power.

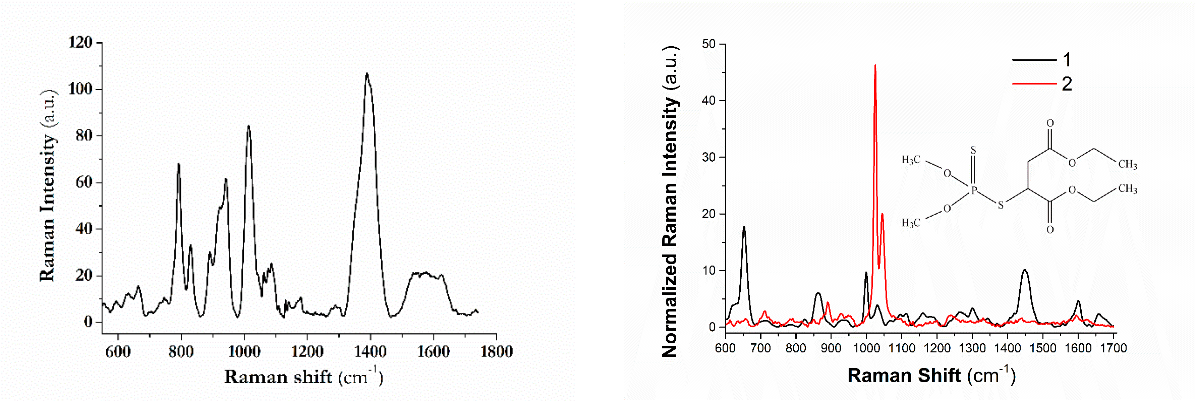

(**a**) (**b**)

Figure S2. (**a**) Raman spectrum of blank rAgNS/AgNS-CF substrate. (**b**) Raman spectrum of 1g/L malathion, measured on a Raman Grade CaF_2 window (1). Raman spectrum of 1 mg/L malathion, measured on the rAgNS/AgNS-CF substrate (2). Insert: the structure of malathion.
